# Multi-Functional Regulation by YAP/TAZ Signaling Networks in Tumor Progression and Metastasis

**DOI:** 10.3390/cancers15194701

**Published:** 2023-09-24

**Authors:** Hannah L. Thrash, Ann Marie Pendergast

**Affiliations:** Department of Pharmacology and Cancer Biology, Duke University School of Medicine, Durham, NC 27710, USA

**Keywords:** YAP/TAZ signaling, tumor metastasis, TEAD-dependent transcription, transcriptional targets, therapeutic strategies

## Abstract

**Simple Summary:**

YAP and TAZ are both transcriptional co-activators in the Hippo pathway. New evidence reveals that they have unique and overlapping functions during tumor progression and metastasis. Here, we discuss current knowledge on YAP and TAZ structure and regulation, document recent findings detailing the role of YAP and TAZ in tumor invasion and metastatic spread, and discuss specific YAP/TAZ targets and therapies. This review underscores the importance of the YAP and TAZ co-activators in cancer metastasis.

**Abstract:**

The Hippo pathway transcriptional co-activators, YES-associated protein (YAP) and Transcriptional Co-Activator with PDZ Binding Motif (TAZ), have both been linked to tumor progression and metastasis. These two proteins possess overlapping and distinct functions, and their activities lead to the expression of genes involved in multiple cellular processes, including cell proliferation, survival, and migration. The dysregulation of YAP/TAZ-dependent cellular processes can result in altered tumor growth and metastasis. In addition to their well-documented roles in the regulation of cancer cell growth, survival, migration, and invasion, the YAP/TAZ-dependent signaling pathways have been more recently implicated in cellular processes that promote metastasis and therapy resistance in several solid tumor types. This review highlights the role of YAP/TAZ signaling networks in the regulation of tumor cell plasticity mediated by hybrid and reversible epithelial–mesenchymal transition (EMT) states, and the promotion of cancer stem cell/progenitor phenotypes. Mechanistically, YAP and TAZ regulate these cellular processes by targeting transcriptional networks. In this review, we detail recently uncovered mechanisms whereby YAP and TAZ mediate tumor growth, metastasis, and therapy resistance, and discuss new therapeutic strategies to target YAP/TAZ function in various solid tumor types. Understanding the distinct and overlapping roles of YAP and TAZ in multiple cellular processes that promote tumor progression to metastasis is expected to enable the identification of effective therapies to treat solid tumors through the hyper-activation of YAP and TAZ.

## 1. Introduction

The YES-associated protein (YAP) and Transcriptional Co-Activator with PDZ Binding Motif (TAZ) are paralog proteins that function as transcriptional co-activators and regulate multiple cellular processes that are critical for solid tumor progression and metastasis [1]. Increased YAP/TAZ expression and activation has been detected in multiple solid tumors including glioblastoma and subtypes of metastatic breast and lung cancer cells. The mechanisms that regulate the activity of YAP/TAZ are diverse and include suppression by the upstream Mammalian Sterile 20-like Kinases 1 and 2 (MST1/2) and the Large Tumor Suppressor homolog 1 and 2 (LATS1/2) protein kinases in the Hippo pathway, as well as Hippo-independent signaling pathways initiated by oncogenic protein tyrosine kinases, G-protein-coupled receptors (GPCRs), adhesion receptors, diverse cellular stress signals, and mechanical forces [2]. Although YAP and TAZ have been thought to be functionally redundant, recent findings demonstrate that these paralog proteins have distinct roles depending on the cellular context, as well as in the response to diverse upstream signals [3,4,5]. Here, we review recent findings on the role of YAP and TAZ in tumor progression to metastasis and highlight the cancer-cell-autonomous roles of YAP and TAZ on tumor cell plasticity, metastatic dissemination, the colonization of distal organ sites, and therapy resistance. We also review the status of current therapies targeting YAP/TAZ signaling to treat distinct tumor types.

## 2. Structure and Regulation of YAP and TAZ

YAP was initially identified as a binding protein and substrate of the YES non-receptor protein tyrosine kinase, a member of the SRC family of tyrosine kinases [6]. TAZ was subsequently identified as a transcriptional co-activator protein that binds to the 14-3-3 adaptor protein [7]. TAZ is encoded by the *WWTR1* gene. The YAP and TAZ paralogs have conserved and unique functions in the regulation of embryonic development, as well as tissue repair and regeneration (reviewed in [8,9]). The dysregulation of YAP and TAZ signaling as a consequence of overexpression and/or enhanced activation downstream of multiple stimuli has a role in metastatic cancer progression and therapy resistance.

### 2.1. YAP and TAZ Structural Domains and Interacting Proteins 

**Figure 1:**
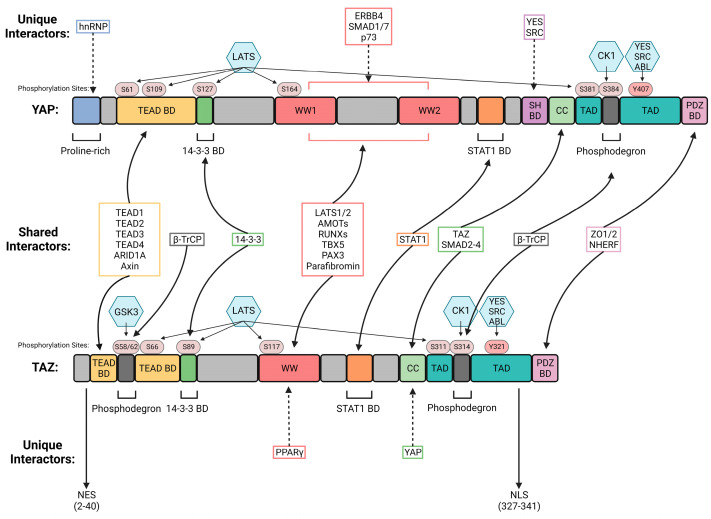
Comparison of YAP/TAZ structure. YAP and TAZ have similar structures with shared domains and interactors. However, they also have unique interactors that contribute to their distinct and individual functions. BD: binding domain; WW1: WW domain; SH: Src homology; CC: coiled coil; TAD: transactivation domain; NES: Nuclear Export Signal; NLS: Nuclear Localization Signal. Created using BioRender.com, accessed on 14 August 2023.

The YAP and TAZ paralog proteins share conserved domains but also have unique sequences that confer the ability to undergo distinct post-translational modifications, as well as the ability to bind common and unique interacting proteins, leading to overlapping or distinct functional properties in diverse tumor types (Figure 1). The three most prominent shared regions of YAP and TAZ are the TEA domain (TEAD) binding domain (TEAD BD), the WW domain, and the transactivation domain (TAD). The TEAD1–4 transcription factors bind to the conserved TEAD BD in the amino (N)-terminus of YAP and TAZ, promoting their transcriptional co-activator function. While YAP and TAZ can equally bind all four TEAD proteins, recent work suggests that TEADs may have distinct transcriptional functions depending on the cellular context. In this regard, TEAD4 upregulation and activity has been reported in diverse tumors (reviewed in [10]). In addition to TEAD1–4, the ARID1A protein was shown to interact with the TEAD BD of both YAP and TAZ (Figure 1). ARID1A, a component of the SWI/SNF complex, functions to inhibit YAP/TAZ-directed transcription by mediating the complex formation of YAP/TAZ with SWI/SNF in the nucleus. This process is regulated by cellular mechano-transduction, and complex formation primarily occurs under conditions of low mechanical strain [1,11].

The WW region of TAZ and YAP is characterized by two distinctive tryptophan residues separated by 20–30 amino acids (Figure 1). Notably, while YAP contains two WW regions, TAZ contains one, which may impact their differential regulation and downstream effector functions. The WW domain mediates binding to LATS1/2, the Hippo pathway serine/threonine protein kinases that phosphorylate YAP/TAZ, leading to proteasomal-mediated degradation. The WW domain also mediates binding to angiomotin (AMOT), which has been shown to bind YAP, sequestering it from the nucleus [12] (Figure 1). In addition to LATS and AMOT, the YAP/TAZ WW1 domains bind to RUNX1/2, T-box transcription factor 5 (TBX5), and paired box gene 3 (PAX3), and promote transcription. The YAP WW sequences have been shown to mediate binding to unique interactors such as ERBB4, SMAD1, and p73. The TAZ WW domain was reported to interact with PPARγ, resulting in co-repressor function in the regulation of insulin sensitivity and general adipocyte biology [13].

Other shared domains present in both YAP and TAZ are the 14-3-3 binding domain, the STAT1 binding domain, the coiled coil (CC) region, and the PDZ binding domain (PDZ BD), which mediate binding to common interactors (Figure 1). The binding of 14-3-3 proteins to serine-phosphorylated sites results in YAP/TAZ sequestration in the cytoplasm, preventing nuclear translocation [7,14]. Other common YAP/TAZ interactors bind to the PDZ region, including zona occludens 1 and 2 (ZO 1/2) and the Na^+^/H^+^ exchange regulatory cofactor (NHERF), which recruit YAP/TAZ to tight junctions and adherens junctions, respectively [12]. These factors sequester YAP/TAZ at intercellular junctions and impair translocation to the nucleus, preventing transcription. The CC domain binds to SMAD2-4 transcription factors, and TAZ binding to the CC domain mediates the formation of a TAZ-YAP heterodimer or a TAZ-TAZ homodimer. YAP can also bind to TAZ through the CC domain, producing a YAP-TAZ heterodimer, however there is no evidence for the formation of a YAP-YAP homodimer [5].

Both YAP and TAZ have phosphodegrons, which are defined as sequences where specific phosphorylation sites promote the binding of a ubiquitin ligase, leading to ubiquitination and degradation [15]. While YAP possesses one phosphodegron in the transactivation domain, TAZ contains two phosphodegrons, one in the N-terminal TEAD BD, and the second one in the C-terminal transactivation domain. CK1 phosphorylates the phosphodegron located in transactivation domain of both YAP and TAZ, and GSK1 phosphorylates the unique phosphodegron site in the TAZ TEAD BD. Upon phosphorylation, β-TrCPs containing E3 ligases bind to the phosphodegrons, leading to ubiquitination and degradation.

Other unique sequences that mediate binding to distinct interactors of the YAP and TAZ paralogs are a proline-rich domain present in YAP alone that can bind heterogeneous nuclear ribonucleoproteins (hnRNP), and binding regions for the kinases YES and Src within the Src homology 3 (SH) binding domain, a domain unique to YAP. Additionally, TAZ possesses defined nuclear export and nuclear localization sequences [16]. The nuclear export sequence lies in the TEAD BD and is masked upon TEAD binding. The nuclear localization sequence is located in the TAZ C-terminus and promotes nuclear uptake upon RhoA activation. Interestingly, the nuclear localization sequence is required for TAZ transcriptional activity [16].

### 2.2. Hippo-Pathway-Mediated Regulation of YAP/TAZ Signaling

**Figure 2:**
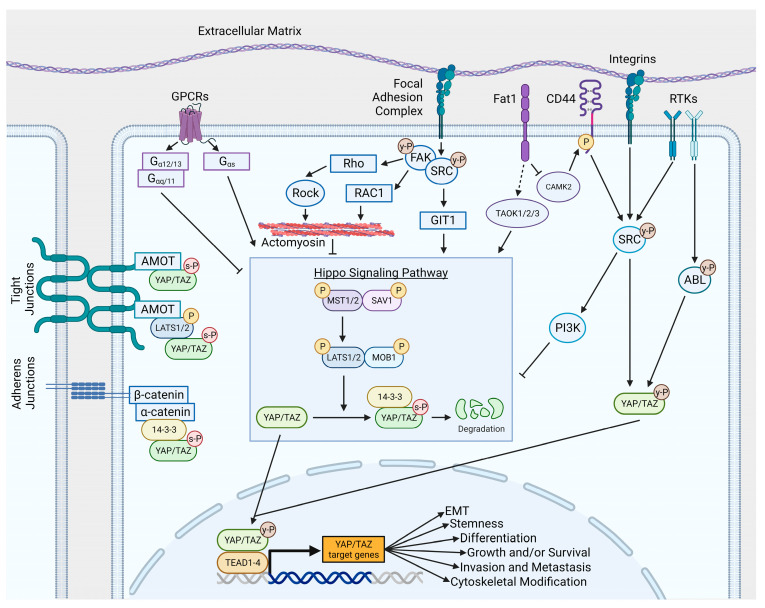
Mechanisms of YAP and TAZ signaling. YAP and TAZ activities are modulated by a number of signaling pathways, leading to the regulation of nuclear and cytosolic YAP/TAZ targets. Created using BioRender.com, accessed on 14 August 2023.

Insight into the regulation of YAP and TAZ functions was first revealed through genetic studies of the Hippo signaling pathway in *Drosophila* (reviewed in [17]). The Hippo pathway regulates organ size and development in *Drosophila* by modulating the activity of Yorkie, the *Drosophila* ortholog of the mammalian YAP and TAZ transcriptional co-activators [18]. To promote growth and survival during development and cancer, YAP and TAZ translocate from the cytosol to the nucleus and bind to transcription factors and/or epigenetic protein complexes [1]. The nuclear localization and function of YAP/TAZ are suppressed by phosphorylation downstream of two sets of serine/threonine protein kinases, MST1/2 and LATS1/2 [19,20,21]. The MST and LATS kinases are evolutionary conserved, core components of the Hippo pathway that suppress the growth-promoting activities of YAP/TAZ in diverse tissues and tumor types (Figure 2). Multiple signals activate the MST kinases, which then recruit and phosphorylate the scaffold protein Salvador (SAV1), and subsequently phosphorylate and activate the LATS kinases with their adaptors MOB1A/B (MOB Kinase Activator 1A and B) (Figure 2). The active LATS kinases bind and phosphorylate YAP and TAZ, thereby preventing nuclear translocation through the formation of cytoplasmic retention complexes or by increasing protein degradation by the ubiquitin proteasome complex. More recently, phosphorylation and activation of core Hippo kinases was shown to occur downstream of the sterile 20-like kinase TAO-1. The TAO-1 kinase was identified in a *Drosophila* genetic screen and shown to regulate the Hippo pathway [22,23]. Additionally, NDR1/2 have also been shown to act as kinases to target YAP, in a manner similar to LATS1/2 [24]. An in-depth description of the Hippo pathway and the various protein kinases that regulate this pathway was given in [25].

Multiple stimuli regulate the activity of the core Hippo kinases and the YAP/TAZ co-activators, including stimuli that regulate cell adhesion and mechanical stress. The FAT1 atypical protocadherin was reported to assemble multiple components of the Hippo signaling complex, including MST1, LATS, MOB1, and Neurofibromin 2 (NF2) leading to YAP inactivation [26]. FAT1 is mutated in glioblastoma and human squamous cell carcinoma (HSCC), including nearly 30% of head and neck squamous cell carcinoma (HNSCC) cases [26,27]. Loss-of-function mutations in *FAT1* and *NF2* can result in enhanced YAP/TAZ protein accumulation, nuclear translocation, and the activation of transcription, leading to tumor progression, metastasis, and therapy resistance. The regulation of transcription by YAP and TAZ in cancer cells is primarily mediated by their interaction with the TEAD family of transcription factors, which bind the DNA of target genes at promoters and enhancers [28] (reviewed in [1]).

Additionally, RASSF1-6 are a family of core Hippo pathway scaffolding proteins that bind MST1/2 in a manner similar to SAV1, leading to the regulation of LATS1/2 and subsequently YAP/TAZ [25,29]. RASSF1A, a member of the RASSF1-6 family, is a known tumor suppressor and has been reported to be hyper-methylated, and thus inactivated, in a number of cancers [30,31]. The RASSF1A protein can be degraded in a TGF-β-dependent manner [32]. RASSF1A plays a role in the apoptotic cascade by promoting MST2 interaction with LATS1 [29]. The MST2-LATS1 axis cross-talks with the ERK pathway to induce apoptosis as well as regulate cell proliferation and cellular transformation [33,34]. Therefore, the inactivation of RASSF1A is considered pro-oncogenic and supports tumor cell survival [29]. Additionally, the silencing of RASSF1A mediates increased the nuclear localization of YAP, leading to enhanced invasion, as well as the regulation of cell stemness and differentiation [35,36]. The re-expression of RASSF1A via DNA methylation inhibitors is under study for its effects on the proliferation, migration, and invasion of cancer cells [37,38,39].

Elevated expression and activation of YAP and TAZ has been detected in many tumor types independently of the genetic inactivation of Hippo pathway components [1]. This observation suggests that in cancer, YAP and TAZ are activated either through Hippo-independent pathways downstream of multiple stimuli and oncogenic proteins in cancer cells, or by alternative inputs, such as the cytoskeleton and cell–cell junctions, which play key roles in YAP/TAZ regulation without genetic or epigenetic alterations [40,41].

### 2.3. Tyrosine Kinases, GPCRs, and Adhesion Receptors Regulate YAP/TAZ Signaling through Hippo-Dependent and Hippo-Independent Pathways

Increased YAP and TAZ protein stability, nuclear translocation, and increased transcriptional activation in cancer cells can occur downstream of multiple oncogenic and cellular stress (i.e., mechanical stress, hypoxia) signals, and can be mediated by activated receptor and non-receptor tyrosine kinases, GPCRs, adhesion receptors, and Rho GTPases through Hippo-dependent and -independent pathways (Figure 2).

While YAP was initially identified as a substrate of the YES non-receptor tyrosine kinase, the role of tyrosine phosphorylation in YAP, TAZ, and core components of the Hippo pathway, is not well understood [6]. The tyrosine phosphorylation of YAP and TAZ has been reported to promote their nuclear localization, protein stability, and/or transcriptional activation. The YES-mediated tyrosine phosphorylation of YAP on Y357 (Y407 in the longer YAP1-2γ isoform) resulted in the formation of a YAP-β-catenin complex with a TBX5 transcription factor that promoted colon cancer cell survival and tumorigenesis [42]. Recently, YES was reported to phosphorylate YAP and TAZ, which was necessary for increased YAP/TAZ nuclear localization and transcriptional activity in hepatocellular carcinoma (HCC) cells and increased liver tumor burden in mice [43]. Elevated YES activity was linked to decreased overall survival in HCC patients.

The SRC non-receptor tyrosine kinase has been shown to activate YAP/TAZ signaling through multiple mechanisms. SRC kinases may directly phosphorylate YAP/TAZ in response to the activation of receptor tyrosine kinases (RTKs), adhesion receptors, and GPCRs among other upstream signals (Figure 2). Epithelial αE-catenin was shown to inhibit the β4 integrin-mediated activation of the SRC kinase, which, in turn, directly phosphorylated YAP on tyrosine sites Y341, Y357, or Y394 (Y391, Y407, and Y444 in the longer YAP1-2γ isoform) within its transcription activation domain, and phosphorylation at these sites was necessary for the SRC–YAP-mediated transformation of skin squamous cell carcinoma (SCC) [44]. The SRC-mediated activation of YAP in confluent keratinocytes was shown to be independent of the canonical Hippo kinases, and the phosphorylation of YAP on Y341/357/394 by SRC was necessary for increased YAP transcriptional activity, nuclear localization, and interaction with TEAD. Alternatively, SRC can activate YAP through several Hippo-dependent mechanisms by repressing the activity of the LATS kinase. SRC was reported to phosphorylate LATS1 directly, inhibiting its kinase activity and leading to YAP activation [45]. Others reported that SRC can repress LATS kinase activity through activation of the phosphatidylinositol 4,5-bisphosphate 3-kinase (PI3K) pathway [46]. More recently, SRC activation was shown to increase YAP/TAZ activity by repressing LATS in breast cancer and melanoma cells through inactivation of GPCR-kinase-interacting protein 1 (GIT1), which, in turn, promotes the LATS-mediated phosphorylation of YAP [47]. SRC activation can indirectly inhibit TAZ protein degradation by decreasing the activity of the SCF (β-TrCP) E3-ligase, thereby blunting TAZ proteasomal degradation, leading to increased TAZ protein levels and the transcriptional activation of target genes [48]. Thus, activated SRC can promote YAP/TAZ activity by targeting diverse pathways depending on the cellular context (Figure 2).

The ABL family of tyrosine kinases, ABL1 and ABL2, have been shown to regulate YAP and TAZ in various cell types. The ABL1 tyrosine kinase was shown to phosphorylate YAP in response to DNA damage, leading to stabilization of the YAP protein, which was dependent on the phosphorylation of YAP Y357 by ABL1 [49]. ABL1 was also reported to phosphorylate murine TAZ on tyrosine Y316 (corresponding to human TAZ Y321) in renal cells following hyperosmotic stress [50]. We have shown that the inhibition of ABL kinases decreases the expression of TAZ in lung adenocarcinoma cells, in part through the regulation of TAZ protein stability [51,52]. Further, we found that the ABL2 tyrosine kinase promoted TAZ nuclear accumulation and transcriptional activation in lung adenocarcinoma cells [52]. The inducible expression of constitutively active ABL2 in lung cancer cells resulted in increased TAZ phosphorylation on tyrosine Y321, which is a predicted ABL SH2 binding site found within the TAZ transactivation domain, and is adjacent to an phosphodegron sequence that mediates recognition by the β-TrCP ubiquitin E3 ligase (Figure 1). We found that endogenous ABL2 interacted with TAZ, but not YAP, in brain metastatic lung cancer cells, and that the interaction of ABL2 with TAZ was markedly decreased by the mutation of TAZ-Y321F. Active ABL2 promoted TAZ tyrosine phosphorylation, leading to enhanced TAZ nuclear accumulation and the transcription of TAZ target genes, including *AXL, ABL2*, and *GAS6* in brain-metastatic lung adenocarcinoma cells [52]. Thus, ABL2 and TAZ engage in bidirectional signaling crosstalk in a feed-forward loop.

The activation of YAP/TAZ has been shown to occur as a consequence of loss-of-function mutations of *FAT1*, which encodes a proto-cadherin through Hippo-dependent and -independent pathways (Figure 2). FAT1 promotes the assembly and activation of Hippo kinases, leading to YAP inactivation [26]. Inactivating mutations in *FAT1* resulting in YAP activation have been detected in HNSCC and other tumors. Alternatively, loss-of-function of *FAT1* was shown to activate YAP independently of Hippo kinases, through the activation of a calcium/calmodulin-dependent kinase 2 (CAMK2), which, in turn, activates SRC through the CD44 cell surface receptor [53]. It was shown that FAT1-mutated tumors exhibited increased sensitivity to SRC and CAMK2 inhibitors, suggesting a potential therapeutic approach to the treatment of these tumors.

GPCRs can activate or inhibit YAP/TAZ signaling depending on the class of GPCR and interaction with distinct heterotrimeric G protein subtypes (Figure 2). Whereas GPCRs coupled to Gαq/11 and Gα12/13 heterotrimeric G proteins inhibit LATS and promote YAP/TAZ nuclear accumulation, GPCRs coupled to Gs activate LATS, thus inhibiting nuclear YAP/TAZ protein accumulation [54]. Activating mutations in the *GNAQ* and *GNA11* oncogenes, encoding for Gα heterotrimeric proteins, have been found in ~90% of uveal melanoma and ~6% of skin melanoma and are associated with YAP nuclear accumulation and drive tumor growth [55,56]. Gαq proteins activate YAP through a Hippo-independent pathway that is mediated by the Trio guanine nucleotide exchange factor, leading to the activation of the RhoA and Rac1 GTPases, which, in turn, promote YAP nuclear translocation and the activation of TEAD-dependent transcription [55]. This finding is in keeping with previous work describing the role of Rho GTPases in YAP/TAZ regulation and activity to modulate cell responses [57,58,59]. Additionally, an analysis of synthetic lethal gene interactions in *GNAQ*-mutated uveal melanoma identified Focal Adhesion Kinase (FAK) as a therapeutic target [60]. Mechanistically, Gαq can also activate FAK by activating the Trio-RhoA pathway, and in turn, FAK regulates YAP activity through the tyrosine phosphorylation of MOB1, thereby disrupting the MOB1/LATS complex, leading to the inhibition of core LATS kinases and YAP activation. Treatment with FAK kinase inhibitors resulted in increased MOB1/LATS interaction, reduced YAP protein levels, and impaired uveal melanoma tumor growth in mice [60]. Thus, Gαq can activate YAP both in a Hippo-independent manner and a Hippo-dependent manner [55,60]. Furthermore, it has been suggested that FAK inhibitors might be exploited as a potential therapy for *GNAQ*-mutated uveal melanoma.

YAP and TAZ can also be activated by metabolites in the mevalonate pathway through the production of geranylgeranyl pyrophosphate (GGPP), which is required for membrane localization and the activation of Rho GTPases, which, in turn, activate YAP/TAZ [61]. The activation of YAP/TAZ by the mevalonate pathway occurs independently of LATS1/2 kinases. It was shown that the inhibition of 3-hydroxy-3-methylglutaryl coenzyme A (HMG-CoA) reductase, the rate-limiting enzyme in the mevalonate pathway, sequesters YAP and TAZ in the cytoplasm of breast cancer cells, leading to decreased YAP/TAZ transcriptional activation, and impaired YAP/TAZ-mediated biological effects such as cell proliferation, cancer stem cell self-renewal, and cell migration [61]. Thus, YAP/TAZ nuclear localization and transcriptional activation in cancer cells can be inhibited indirectly by targeting upstream regulators that are amenable to pharmacological intervention.

## 3. YAP and TAZ Promote Tumor Progression and Metastasis through Dysregulation of Diverse Cellular Processes

The enhanced activation of YAP and TAZ in tumors has been shown to promote the growth and metastasis of multiple tumor types. The activation of YAP and TAZ signaling often occurs independently of genetic alterations (reviewed in [1]). However, genetic amplifications of *YAP1* and/or *WWTR1* have been detected in cervical, ovarian, HNSCC, and esophageal squamous tumors (reviewed in [1,62]). Notably, targeted DNA sequencing of lung adenocarcinoma tumors revealed that Hippo pathway alterations were associated with shorter time to emergence of brain metastasis [63]. In this regard, *YAP1* amplification was reported to occur at a higher frequency in lung adenocarcinoma brain metastases compared to primary tumors [64].

Some solid tumors exhibit genetic alterations in upstream components of the Hippo pathway, leading to YAP/TAZ activation. Among these are *NF2* mutations in mesothelioma, meningioma, medulloblastoma, schwannoma, and renal cell carcinoma. Recently, *FAT1* deletions or protein-truncating nonsense mutations have been found in HNSCC and other human SCC tumors, leading to YAP/TAZ activation [26,53]. In rare tumors, the oncogenic activation of TAZ and YAP can occur as a consequence of chromosomal alterations that generate gene fusions, leading to the expression of chimeric proteins such as TAZ-CAMTA1 and YAP-TFE3 that drive epithelioid hemangioendothelioma (EHE) in humans and mouse models [65,66,67,68].

The enhanced expression and activation of YAP and TAZ without the inactivation of Hippo pathway components has been detected in a wide range of tumors, particularly in metastatic and therapy-resistant tumors [52,69,70]. The increased expression of YAP and TAZ in these tumors has been shown to occur as a consequence of the hyper-activation of diverse oncogenic signals including tyrosine kinases, GPCRs, and metabolic pathways (reviewed in [2,61]).

A direct role for YAP in promoting cancer metastasis was first reported by Lamar et al. [28], who showed that that active YAP promoted the metastasis of breast cancer and melanoma cells, and that the YAP TEAD binding domain was required for tumor progression and metastasis. Subsequent work demonstrated that YAP and/or TAZ promoted metastasis in multiple tumor types by regulating distinct steps in the metastatic cascade (reviewed in [2]).

**Figure 3:**
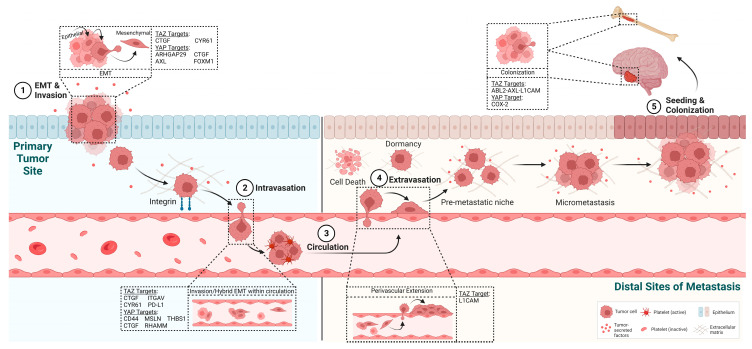
Role of YAP/TAZ in the metastatic cascade. YAP and TAZ have varied roles in the tumor progression of metastasis and regulate the activity of multiple transcriptional targets implicated in distinct steps in the metastatic cascade. Created using BioRender.com, accessed on 14 August 2023.

While it is well known that YAP and TAZ can regulate cancer cell growth, survival, migration, and/or invasion depending on the cellular context and tumor type, recent reports have implicated YAP/TAZ signaling in the regulation of tumor cell plasticity mediated by hybrid and/or reversible EMT states and the promotion of cancer stem cell/progenitor phenotypes (Figure 3).

### 3.1. Role of YAP/TAZ in the Regulation of Reversible EMT, Migration, and Invasion

The initiation of epithelial tumor metastasis requires an epithelial-to-mesenchymal transition, a process that promotes the loss of epithelial cell polarity, the disruption of cell–cell adhesions, the acquisition of mesenchymal characteristics, detachment from the primary tumor, and increased migration and invasion [71] (Figure 3). Notably, the EMT process is also associated with the emergence of cancer stem cells (CSCs), tumor cell plasticity, and resistance to therapy [71,72]. Further, accumulating data has shown that EMT is not a binary process, but rather, is characterized by hybrid or partial EMT states that express different levels of epithelial and mesenchymal markers [72]. Moreover, EMT is a reversible process as metastatic tumors cells undergo a mesenchymal-to-epithelial transition (MET) to colonize distal organs sites (Figure 3).

The enhanced expression and activation of YAP/TAZ were shown to promote EMT processes such as the dissolution of epithelial cell–cell junctions, the expression of mesenchymal markers, and the acquisition of mesenchymal cell morphology, in part, through the activation of EMT-inducing transcription factors such as SNAIL1, SLUG, TWIST, and ZEB1 [73,74,75]. YAP was shown to function together with KRAS in cancer cells dependent on KRAS for viability to promote not only cell survival but also EMT [76]. YAP interacts with the FOS transcription factor at the promoters of EMT genes such as *SNAIL2* in KRAS-driven cancer cells [76]. Recently, it was shown that loss-of-function of *FAT1*, a gene that encodes a proto-cadherin cell surface receptor, activates a CAMK2-CD44-SRC signaling pathway that induces YAP nuclear translocation and activation, leading to ZEB1 expression (Figure 2), which, in turn, promotes a hybrid EMT state, and increases metastasis in squamous cell carcinoma [53]. The induction of EMT in the primary tumor is followed by enhanced migration and invasion, which allow for the dissemination to distal organ sites of metastasis (Figure 3). A role for YAP and TAZ in the migration and invasion of multiple cancer cell types has been reported, and in some studies, these phenotypes have been shown to be dependent on the binding of YAP/TAZ to the TEAD transcription factors [77,78,79].

### 3.2. Role of YAP/TAZ in the Regulation of Tumor–Endothelial Cell Interactions: Entry and Exit from the Vasculature and Intravascular Motility

Following release from the primary tumor, invasive cancer cells must enter into the vasculature through the process of intravasation, and subsequently exit from the circulation via extravasation. While few reports have implicated YAP/TAZ in the regulation of intravasation [80], several studies have demonstrated a role for YAP and TAZ in the extravasation of cancer cells. We reported that the extravasation of lung adenocarcinoma cells in a mouse model of metastases required the ABL kinase-mediated activation of TAZ signaling [51]. YAP was shown to promote the extravasation of breast cancer cells, in part, through increased cytokine expression [81]. Intravital imaging studies revealed that the expression of constitutively active YAP promoted the intravascular motility of A375 melanoma cells and increased metastatic spread without altering tumor cell extravasation, survival in the circulation, or proliferation [82]. Thus, the role for YAP/TAZ in the regulation of extravasation and intravascular motility is likely dependent on the tumor type and the tumor microenvironment.

YAP/TAZ have various roles in shaping the tumor microenvironment (TME) to facilitate intravascular motility and general tumor promotion. YAP/TAZ were shown to actively contribute to the remodeling of the TME by promoting angiogenesis, mediating mechano-transduction, and stimulating metabolic reprogramming, among various cellular processes [83,84]. YAP and TAZ also play a role in the immune system during tumor progression [85,86]. The roles of YAP/TAZ in cancer immunity are an emerging field of study and have been reviewed elsewhere [83,84,87]. It is worth mentioning, however, the role played by YAP/TAZ in cancer-associated fibroblasts. Cancer-associated fibroblasts have, in general, pro-oncogenic functions as their activity serves to promote angiogenesis, influence the immune environment, and facilitate metabolic changes [88]. In cancer-associated fibroblasts, YAP activation contributes to a feed-forward loop by which YAP activity regulates the actin cytoskeleton, which further promotes YAP activity to enhance tumor progression [89]. YAP function has been shown to be essential to the role of cancer-associated fibroblasts in supporting oncogenic phenotypes [89,90].

### 3.3. Role of YAP/TAZ in Seeding and Colonization

The seeding and colonization of distal organ sites are rate-limiting steps of the metastasis cascade [91]. YAP and TAZ have been shown to regulate these processes, leading to the metastatic outgrowth of disseminated cancer cells. A role for the L1CAM (cell adhesion molecule 1)-mediated activation of YAP was shown to be required for the metastatic seeding and colonization of lung adenocarcinoma and breast cancer cells [92]. Following extravasation, the activation of L1CAM-YAP signaling promotes the spreading of disseminated cancer cells (DCCs), displacing resident pericytes on the abluminal surface of blood vessels, leading to metastatic outgrowth. YAP activation has been shown to promote metastasis by enhancing the survival and proliferation of breast cancer and melanoma cells [28]. TAZ activation downstream of activated ABL kinases in triple-negative breast cancer cells was shown to promote metastasis to the bone through the regulation of tumor–bone-niche interactions [93]. YAP activation in breast cancer cells has also been shown to promote metastasis to the bone, but through the regulation of osteoclast differentiation [94]. Thus, the inhibition of YAP/TAZ signaling could be effective in preventing the metastatic seeding and colonization of distal sites by DCCs. In this regard, the allosteric inhibition of ABL kinases induced a profound decrease in metastasis to the bone in mice bearing triple-negative breast cancer cell tumors [93].

A TAZ-AXL-ABL2 autocrine signaling axis was shown to be required for lung adenocarcinoma brain metastasis [52]. Effective therapies to treat brain metastasis are lacking due, in part, to the presence of the blood–brain barrier (BBB), which prevents the accumulation of anti-tumor drugs in the brain, and/or the emergence of therapy resistance. Notably, the treatment of mice bearing brain metastases with allosteric inhibitors of ABL tyrosine kinases, which are BBB-penetrant, impaired metastasis by decreasing the seeding and colonization of EGFR mutant lung adenocarcinoma cells in the brain parenchyma [52].

### 3.4. YAP/TAZ Promote Therapy Resistance and Acquisition of Cancer Stem Cell Phenotypes, Leading to Enhanced Metastasis

Increased YAP/TAZ expression and activation have been linked to resistance to chemotherapy, radiation, and targeted therapies for the treatment of diverse tumor types [69,70,95,96,97,98,99]. For example, enhanced TAZ expression was shown to enhance the resistance of HNSCC cells to chemotherapy [75], and YAP was reported to mediate HNSCC resistance to the MEK inhibitor trametinib [100]. Recently, an allosteric pan-TEAD inhibitor, GNE-7883, was shown to be effective in overcoming resistance to the KRAS G12C inhibitor sotorasib in mouse models [101].

The emergence of therapy resistance in cancer cells can be induced by phenotypic plasticity and the acquisition of cancer stem cell phenotypes (reviewed in [71]). The activation of TAZ or YAP can induce cancer cell plasticity and the conversion of differentiated cells into somatic stem cells [102]. In this regard, the deletion of *Taz* and *Yap1* in a mouse SCC model induced by *Fat* knockout decreased tumor stemness and metastasis [53]. Further, YAP and TAZ were shown to promote stemness and cell plasticity in glioblastoma (GBM) [103]. YAP/TAZ activation induced GBM stem-cell-like phenotypes and prevented the differentiation of cancer stem cells along the neuronal lineage. Moreover, YAP/TAZ knockout cells were defective for tumor initiation and maintenance in GBM mouse models. The inhibition of YAP/TAZ signaling was reported to overcome resistance to the BRAF inhibitor vemurafenib and decrease melanoma stem cell survival [104]. A greater mechanistic understanding of the YAP/TAZ-mediated regulation of tumor cell plasticity is needed to overcome therapy resistance in multiple tumor types.

## 4. Transcriptional and Epigenetic Targets of YAP and TAZ in Cancer Cells

While numerous YAP/TAZ transcriptional targets have been identified using loss- and gain-of-function screens, only a fraction of these targets have been validated via in vitro and in vivo functional assays. Here, we discuss a panel of YAP/TAZ direct transcriptional targets that play a role in tumor progression to metastasis, and that have been validated using functional assays (Table 1).

Activated TAZ in lung adenocarcinoma cells elicits the expression of a panel of target genes that promote brain metastasis, including *ABL2*, *AXL*, and *L1CAM* [52]. Activated TAZ was shown to bind to the *ABL2* promoter through the use of ChIP-qPCR. The increased production of *ABL2* creates a positive feedback loop whereby ABL2 activates TAZ and promotes the increased transcription of both *AXL* and *L1CAM*. The TAZ-ABL2-AXL signaling axis promotes the increased expression of a panel of brain metastatic targets, including *L1CAM*, required for lung adenocarcinoma brain metastasis. Treatment with the ABL kinase inhibitor ABL001 impairs the expression of TAZ targets and decreases the outgrowth of brain metastases [52]. Further, L1CAM activity in disseminated cancer cells is required for perivascular spreading, or extension, and induces the activation of YAP, which, in turn, promotes the outgrowth of metastasis-initiating cells. L1CAM-YAP signaling was shown to be necessary for metastatic colonization in multiple organs [92]. Thus, L1CAM can function both upstream and downstream of YAP/TAZ signaling in cancer cells.

*AXL* expression is also regulated by YAP/TAZ signaling independently of ABL kinases. The *AXL* promoter contains four TEAD binding sites, and YAP binds to the *AXL* promoter region in hepatocellular carcinoma [106]. In osteosarcoma, AXL has been linked to YAP/TAZ signaling and promotes cell stemness, leading to enhanced metastasis [120]. YAP-driven resistance to targeted therapies in non-small-cell lung cancer can be mediated, in part, by enhanced AXL expression, which has been shown to be YAP-dependent, and the inhibition of AXL kinase activity results in restored drug sensitivity [121]. Further, in melanoma, AXL promotes invasion and metastasis and is a marker of poor prognosis [122]. Therefore, AXL plays diverse roles in promoting invasion, metastasis, and drug resistance linked to YAP signaling.

Thrombospondin 1 (*THBS1* encoding the TSP1 protein) regulates cell adhesion and is a YAP target gene [119]. TSP1 has a role in promoting FAK phosphorylation and activation. Active FAK enhances the mobility and invasiveness of tumor cells and regulates various matrix metalloproteases, which promote invasive cell behavior [123]. TSP1 has been shown to mediate invasion in melanoma models [122]. Similarly, *ITGAV*, which is a known TAZ target gene encoding integrin-αV, has been shown to promote cell mobility and is overexpressed in various solid tumors, such as bladder, colorectal, prostate, and breast cancers [124]. In hepatocellular carcinoma, the TAZ/TEAD complex binds the *ITGAV* promoter to induce its expression [114].

Connective tissue growth factor (CTGF) is a secreted protein encoded by *CTGF,* a direct YAP target gene, as shown by the binding of the YAP-TEAD complex to the *CTGF* promoter using ChIP assays [109]. *CTGF* has also been shown to be a direct TAZ transcriptional target [110]. The related *CYR61* (*CCN1*) is also a TAZ target gene [112]. Both CTGF and CYR61 are members of the connective tissue factor family of proteins that can function as integrin ligands and regulate cell proliferation, apoptosis, cell migration, and angiogenesis depending on the cellular context. These proteins can function to promote tumor progression and have been linked to the Hippo pathway through YAP and TAZ [111,112]. CTGF activity in breast cancer leads to enhanced migration [125]. Both CTGF and CYR61 work together to promote drug resistance in breast cancer [112]. Like THBS1, CYR61 has also been directly implicated in the promotion of melanoma metastasis by increasing cell invasion [122]. In osteosarcoma, CYR61 is responsible for inducing EMT and promoting metastatic invasion to the lung [126].

The FOXM1 transcription factor has multiple roles in cancer progression and metastasis, including promoting cell proliferation, self-renewal, migration, invasion, angiogenesis, and EMT [127]. The YAP/TEAD4 complex binds the *FOXM1* promoter, leading to its upregulation [113]. The inhibition of FOXM1 in cancer cells results in decreased cell proliferation and migration, impaired metastasis, and reduced drug resistance (reviewed in [128,129]). Upon FOXM1 upregulation, other factors, including cytoskeletal proteins, are also upregulated, leading to a drastic overhaul of the cellular keratin filament network and increased metastatic spread. FOXM1 activity has been implicated in various cancers, including breast, lung, and colorectal, and correlates with poor prognosis [130,131].

The expression of another YAP target, ARHGAP29 (Rho GTPase-activating protein 29), has been shown to correlate with the metastatic potential of several cancers [105]. Increased ARHGAP29 expression causes a distinct change in actin dynamics and leads to the depolymerization of F-actin into G-actin [105]. This change creates a softening of the cytoskeleton and promotes cell migration. 

Mesothelin (MSLN) is a differentiation antigen present on mesothelial cells that is overexpressed in various cancers, including ductal pancreatic, ovarian, and lung tumors, and has been used as a tumor biomarker and target for treatment [132,133]. MSLN has been linked to YAP through YAP/TEAD1 binding to the Canscript sequence on its promoter region [115]. YAP and MSLN are co-expressed in fibrolamellar carcinoma, and have been suggested to be potential therapeutic targets for the treatment of this tumor type [134]. MSLN promotes cancer cell invasion and migration by increasing MMP-7 expression, leading to ECM degradation mediated by the MAPK/ERK and JNK signaling pathways [135]. MSLN can also promote MMP-7 expression by binding to Mucin 16 in pancreatic cancers [136].

*CD44* and *RHAMM* are YAP target genes that, when upregulated, also promote cell invasion [107,118]. CD44 and RHAMM both bind hyaluronic acid and together are involved in ERK1/2 regulation. Each of these activities leads to increased cancer cell invasion potential [132,137].

Other targets of YAP and TAZ include *COX-2* and *PD-L1*. COX-2 (Cyclooxygenase 2) is involved in drug resistance in many cancer types and is activated directly by YAP in colorectal cancer [108]. The cascade activated by the YAP-mediated transcription of *COX-2* leads to an increase in cell proliferation and colony forming ability [108]. *PD-L1* (*CD274*) is expressed in cancer cells and the PD-L1 protein mediates the interaction between cancer cells and T cells, leading to the suppression of T cell activity and immune evasion by cancer cells. It was reported that PD-L1 expression is regulated by TAZ activity in breast cancer cells through TAZ binding to the *PD-L1* promoter region [117]. This finding links the Hippo pathway to the promotion of immune invasion in cancer cells.

It is interesting to note that a subset of genes are targets of either TAZ or YAP, but not both. This finding underscores the notion that YAP and TAZ are not identical, and while they share overlapping functions, they have unique and divergent functions [5]. More work is needed to elucidate the specific transcriptional programs regulated by these two factors. For example, work by Zanconato et al. has described a set of TAZ and YAP transcriptional targets in breast cancer cells that includes *MYC*, a cell cycle regulator and known oncogene [116]. However, additional studies are needed to clarify the link between YAP/TAZ and MYC. While multiple TAZ and YAP targets have been described (reviewed in [132]), there is a gap in our current understanding of their diverse roles in the metastatic cascade in various tumor types.

## 5. Therapeutic Strategies for Targeting YAP/TAZ Signaling in Metastatic and Therapy-Resistant Tumors

As YAP and TAZ play important and varied roles in tumor progression, they have become promising targets for cancer treatment. It was thought that YAP and TAZ, as transcription factors, were undruggable targets [1,138]. However, due to recent advances in the molecular understanding of the structure and function of these transcription factors and their interacting partners, many promising treatment options are being explored that target YAP/TAZ signaling in tumors. Some of these promising strategies are described below (Table 2).

### 5.1. Targeting YAP/TAZ and TEAD Expression and Interaction

A new therapy directly targeting YAP/TAZ expression is ION537. ION537, from Ionis Pharmaceuticals, is a new class of drug that works as an antisense oligonucleotide [149]. It targets *YAP1* mRNA and inhibits its translation, depleting YAP expression by up to 90% in models of hepatocellular cancer [139]. It has also been shown to deplete YAP levels in tumor xenografts, leading to a noted decrease in tumor growth. ION537 is currently in clinical trials.

Unlike ION537, which targets YAP expression, other therapies target the interaction between YAP/TAZ and TEAD, in some cases by directly targeting TEAD to disrupt its interaction with YAP/TAZ. The classic drug in this category is verteporfin, the first therapy discovered to inhibit YAP/TAZ and TEAD binding [140]. Verteporfin is a member of the porphyrin family and works effectively in vitro to block YAP/TAZ and TEAD interaction. However, verteporfin exhibits low potency in vivo as a treatment for tumors, and therefore, it is mainly used in vitro to disrupt the YAP/TAZ and TEAD interaction, though its mechanism of action is unclear and remains to be defined [140,150,151]. There are several clinical trials underway using verteporfin, as it is FDA-approved in combination with light to treat eye diseases; only one of these current trials is for cancer therapy focusing on EGFR-mutated glioblastoma, and it remains unclear whether verteporfin’s effects are mediated through disruption of the YAP/TAZ and TEAD interaction.

Another first-in-class therapy from Novartis is IAG933. IAG933 is a small molecule and acts as a true YAP/TAZ and TEAD binding inhibitor by binding to the surface of TEAD, impeding the ability of YAP to bind [141]. It is unknown whether it has been specifically tested with TAZ; however, it is currently in clinical trials.

Flufenamic acid is a commercially available NSAID (Non-Steroidal Anti-Inflammatory Drug) that was identified as a small molecule inhibitor of YAP/TAZ and TEAD binding [142,143]. Though its mechanism of action is not fully elucidated, flufenamic acid may function as an auto-palmitoylation inhibitor of TEAD by binding to the TEAD central pocket, the region which also binds palmitate [143,152]. TEAD must be palmitoylated in order to bind YAP/TAZ and has been shown to auto-palmitoylate; therefore, inhibiting its ability to bind palmitate effectively or auto-palmitoylate inhibits its YAP/TAZ binding ability [153]. There is some evidence to show that flufenamic acid may also act as an allosteric protein–protein interaction disruptor between YAP/TAZ and TEAD, though this remains to be definitely proven [152].

Another compound that functions as an auto-palmitoylation inhibitor of TEAD is VT3989 (Vivace Therapeutics). This compound targets the TEAD hydrophobic, palmitate binding pocket, thereby preventing palmitoylation [144,154]. VT3989 is currently in clinical trials. Vivace Therapeutics has also developed a number of other compounds that are effective in preclinical tumor models and that have been previously reviewed [155].

Other auto-palmitoylation inhibitors of TEAD include K-975 [145,152,156], MGH-CP1 [146], and IK-930 [157]. All three compounds bind the central pocket of TEAD, effectively blocking TEAD palmitoylation and YAP/TAZ binding. Of note, IK-930 has only been shown to work effectively in Hippo pathway-deficient or dysregulated cells and is currently in clinical trials. Other potential protein–protein interaction disruptors for YAP/TAZ-TEAD binding are still under development [152].

Recently, an allosteric pan-TEAD inhibitor, GNE-7883, was reported to block YAP/TAZ interactions with all four TEAD paralogs by binding to the TEAD lipid pocket [101]. Treatment with GNE-7883 suppressed the expression of YAP/TAZ target genes, decreased the cell proliferation of several cell lines in vitro, and impaired xenograft growth in mice [101]. Further, GNE-7883 treatment was effective in overcoming both intrinsic and acquired resistance to the KRAS G12C inhibitor sotorasib in preclinical mouse models [101]. Despite these encouraging results, it was reported that GNE-7883 has suboptimal pharmacokinetic properties and low oral bioavailability, and therefore, GNE-7883 had to be administered subcutaneously. Derivative compounds with improved oral bioavailability and pharmacokinetic properties are needed to treat YAP/TAZ/TEAD-dependent tumors.

### 5.2. Therapies Targeting YAP/TAZ Regulatory Factors

YAP/TAZ activity can be inhibited indirectly by targeting factors used by YAP/TAZ to carry out their cellular functions or factors upstream of YAP/TAZ. Bromodomain-containing protein 4 (BRD4) interacts with YAP/TAZ and is recruited to the chromatin to potentiate YAP/TAZ-mediated transcription. Therefore, inhibiting BRD4 may be an effective way to inhibit YAP/TAZ activity [158]. Bromodomain and extra-terminal domain (BET) inhibitors, which effectively inhibit BRD4, have been shown to suppress tumorigenesis in vivo and show promise in preclinical models. However, in the many clinical trials involving BET inhibitors, reviewed in [147], the results to date are inconclusive. AP-1 is also recruited to the chromatin by YAP/TAZ [116]. T-5224 is an AP-1 inhibitor that has been shown to work effectively in head and neck cancers, though the anti-tumor effects of T-5224 have not yet been linked to YAP/TAZ inhibition [159].

Several kinases and Rho-GTPases function upstream to activate YAP/TAZ. Using inhibitors to target these factors can be exploited to decrease YAP/TAZ activity, but their cellular effects may not be restricted to the inhibition of YAP/TAZ signaling alone. SRC is one of the upstream kinases that promote YAP/TAZ activation. Treatment with dasatinib, a small-molecule inhibitor targeting SRC and multiple other kinases, has been shown to inhibit YAP/TAZ in vitro and in vivo (reviewed in [2,47]). Clinical trials with dasatinib, however, show inconclusive results, and it is uncertain whether any tumor responses are due to YAP/TAZ inhibition [1]. ABL kinases can also function upstream of YAP/TAZ, leading to increased nuclear localization and activation. Treatment with asciminib (ABL001), an allosteric ABL kinase inhibitor, has been shown to impair TAZ activity, as measured by a profound decrease in TAZ-dependent transcription. TAZ protein stability is also affected by ABL kinases. ABL kinases function to stabilize TAZ in cancer cells, and their inhibition leads to subsequent protein degradation [51]. ABL kinases can promote TAZ nuclear localization, Ref. [52] and ABL kinase activity has been reported to antagonize YAP function as well [160]. Finally, the functional regulation of YAP/TAZ by the Rho-kinase pathway (ROCK), has led to the use of ROCK inhibitors as a means of inhibiting YAP/TAZ function. These inhibitors are reported to be effective in vitro and in vivo [161].

## 6. Conclusions and Future Directions

Much is known about the structure and regulation of the Hippo pathway co-activators YAP and TAZ. Recent advances have been made in YAP/TAZ research, including the identification of cellular processes and transcriptional targets directly affected by YAP and TAZ, some of which are discussed here. Notable are the development of therapeutic strategies to target and inhibit YAP/TAZ signaling and their oncogenic functions during tumor progression and metastasis. However, a major challenge for researchers is the identification of biomarkers to distinguish which patients would benefit from YAP/TAZ targeting therapies, versus those that would not. Further work is needed to elucidate the emerging roles of YAP and TAZ, including exciting new research on the roles of YAP and TAZ in the epigenetic regulation of target genes [162]. Moreover, a greater understanding is needed to define the roles of chromatin and histone modification in the regulation of YAP and TAZ, and their downstream transcriptional pathways. It has been noted that YAP/TAZ and TEAD binding complexes often bind to enhancer regions on the chromatin [163]. The differences in the expression and activity that arise due to enhancer binding or promoter binding by YAP/TAZ complexes is an area of active research. Finally, while YAP and TAZ are related proteins with overlapping functions, they also have unique roles [3,4,5]. To fully understand the roles YAP and TAZ play in tumor progression and metastasis, it is important to first clarify their individual roles in order to determine how these two factors function to regulate cancer progression and metastasis in various tumor types.

## Figures and Tables

**Table 1:** Selected validated YAP/TAZ transcriptional targets involved in metastasis regulation.

Target Gene	Upstream Signaling Molecule	ChIP-qPCR	TEAD Binding Sites	Mechanism
*ABL2*	TAZ	[52]	Yes [52]	Seeding Colonization
*ARHGAP29*	YAP	[105]	Yes [105]	Cell Motility Invasion
*AXL*	YAP TAZ [52]	[106]	Yes [52,106]	Invasion Stemness Seeding Colonization Therapy Resistance
*CD44*	YAP	[107]	Yes [107]	Cell Motility Invasion
*COX-2*	YAP	[108]	Yes [108]	Cell Proliferation Colonization
*CTGF*	YAP TAZ	YAP [109] TAZ [110]	Yes [111]	Cell Motility Invasion Therapy Resistance
*CYR61*	TAZ	[112]	Yes [111]	Cell Invasion EMT Therapy Resistance
*FOXM1*	YAP	[113]	Yes [113]	Cell Proliferation EMT Invasion
*ITGAV*	TAZ	[114]	Yes [111]	Cell Motility Invasion
*L1CAM*	TAZ	[52]	Yes [52]	Seeding Colonization Perivascular Extension
*MSLN*	YAP	[115]	Yes [115]	Cell Motility Invasion
*MYC*	YAP/TAZ	[116]	Yes [116]	Cell Growth
*PD-L1*	TAZ	[117]		Immune Evasion
*RHAMM*	YAP	[118]	Yes [118]	Cell Motility Invasion
*THBS1*	YAP	[119]	Yes [119]	Cell Motility Invasion

**Table 2:** Therapeutic strategies for targeting YAP/TAZ.

Drug	Source (Company)	Target	Preclinical Studies	Clinical Trials
ION537	Ionis Pharmaceuticals	Anti-YAP Antisense Oligonucleotide	[139]	Phase 1 Completed NCT04659096 (Advanced solid tumors)
Verteporfin (Visudyne)	Novartis	YAP/TAZ and TEAD interaction inhibitor	[140]	Phase 1/2 Recruiting NCT04590664 (EGFR-mutated glioblastoma)
IAG933	Novartis	YAP/TAZ and TEAD interaction inhibitor	[141]	Phase 1 Recruiting NCT04857372 (Mesothelioma)
Flufenamic acid	Commercially Available	TEAD palmitoylation inhibitor YAP/TAZ and TEAD interaction inhibitor	[142,143]	--
VT3989	Vivace Therapeutics	TEAD palmitoylation inhibitor YAP/TAZ and TEAD interaction inhibitor	[144]	Phase 1 Recruiting NCT04665206 (Mesothelioma)
K-975	Kyowa Kirin (Tokyo, Japan)	TEAD palmitoylation inhibitor YAP/TAZ and TEAD interaction inhibitor	[145]	--
MGH-CP1	Commercially Available	TEAD palmitoylation inhibitor YAP/TAZ and TEAD interaction inhibitor	[146]	--
IK-930	Ikena Oncology	TEAD palmitoylation inhibitor YAP/TAZ and TEAD interaction inhibitor	--	Phase 1 Recruiting NCT05228015 (Epithelioid hemangioendothelioma and mesothelioma)
GNE-7883	Genentech	Allosteric pan-TEAD Inhibitor	[101]	--
BET inhibitors	Multiple	BRD4 Inhibitor	Numerous Reviewed in [147]	Numerous Reviewed in [147]
Dasatinib	Sprycel	SRC Inhibitor	Numerous Reviewed in [148]	Numerous Reviewed in [148]
Asciminib (ABL001)	Novartis	ABL Inhibitor	[52]	--

## Data Availability

The data are contained within the article.

## Abbreviations

ABL1 (c-ABL) and ABL2 (Arg)	Abelson Murine Leukemia Viral Oncogene Homologs 1 and 2
AMOT	Angiomotin
ARHGAP29	Rho GTPase-Activating Protein 29
BBB	Blood–Brain Barrier
BET	Bromodomain and Extraterminal Domain
BRD4	Bromodomain-Containing Protein 4
CAMK2	Calcium/Calmodulin-Dependent Kinase 2
CC	Coiled Coil Domain
COX-2	Cyclooxygenase 2
CSC	Cancer Stem Cells
CTGF	Connective Tissue Growth Factor
DCC	Disseminated Cancer Cells
EHE	Epithelioid HemangioEndothelioma
EMT	Epithelial–Mesenchymal Transition
ERK	Extracellular Signal-Regulated Kinases
FAK	Focal Adhesion Kinase
GBM	Glioblastoma
GGPP	Geranylgeranyl pyrophosphate
GIT1	GPCR-kinase-Interacting Protein 1
GPCRs	G-Protein-Coupled Receptors
HMG-CoA	3-hydroxy-3-methylglutaryl coenzyme A
hnRNP	Heterogeneous Nuclear Ribonucleoproteins
HNSCC	Head and Nek Cancer Squamous Cell Carcinoma
L1CAM	Cell Adhesion Molecule 1
LATS 1/2	Large Tumor Suppressor Homologs 1 and 2
MET	Mesenchymal–Epithelial Transition
MOB 1A/B	MOB Kinase Activator 1A and B
MSLN	Mesothelin
MST 1/2	Mammalian Sterile 20-Like Kinase 1 and 2
NDR1/2	Nuclear Dbf2-Related Kinase 1 and 2
NES	Nuclear Export Signal
NHERF	Na^+^/H^+^ Exchange Regulatory Cofactor
NLS	Nuclear Localization Signal
NSAID	Non-Steroidal Anti-Inflammatory Drug
PAX3	Paired Box Gene 3
PDZ BD	PDZ Binding Domain
PI3K	Phosphatidylinositol 4,5-bisphosphate 3-kinase
RASSF1-6	Ras Association Domain Family Members 1-6
RASSF1A	Ras Association Domain Family Protein 1 Isoform A
ROCK	Rho-Kinase Pathway
SAV1	Salvador
SH BD	Src Homology Binding Domain
TAD	Transactivation Domain
TAZ	Transcriptional Co-Activator with PDZ Binding Motif
TBX5	T-box Transcription Factor 5
TEAD BD	TEAD Binding Domain
TGF-β	Transforming Growth Factor-β
TME	Tumor Microenvironment
TSP1	Thrombospondin 1
YAP	YES-Associated Protein
ZO 1/2	Zona Occludens 1 and 2
